# Breaking the Habit: A Systematic Review and Meta-Analysis of Pregnancy-Related Smoking Cessation Randomized Controlled Trials

**DOI:** 10.3390/healthcare13070732

**Published:** 2025-03-26

**Authors:** Omnia S. Elseifi, Faten Ezzelarab Younis, Iman M. Mirza, Abdullah Alhewiti, Nahla M. S. Abd-Elhady, Eman M. Mortada

**Affiliations:** 1 Department of Family and Community Medicine, Faculty of Medicine, University of Tabuk, Tabuk 47913, Saudi Arabia; oelseifi@ut.edu.sa (O.S.E.); fa.younes@ut.edu.sa (F.E.Y.); imirza@ut.edu.sa (I.M.M.); aalhewiti@ut.edu.sa (A.A.); 2 Department of Pediatric, College of Medicine, King Faisal University, Alahsa 31982, Saudi Arabia; nabdelgawad@kfu.edu.sa; 3 Family and Community Medicine Department, College of Medicine, Princess Nourah bint Abdulrahman University, P.O. Box 84428, Riyadh 11671, Saudi Arabia

**Keywords:** smoking, pregnancy, behavioral approaches, pharmacological therapies, RCT

## Abstract

**Background:** Smoking during pregnancy is a significant issue because of its impact on maternal and fetal health. This study aimed to ascertain the effects of smoking cessation programs on the smoking abstinence rate and pregnancy outcomes. **Methods:** A meta-analysis of twenty-one RCTs was carried out in accordance with PRISMA standards. The meta-analysis comprised 8149 pregnant smokers. With RevMan (version 5.4), the pooled effect of RR for different smoking cessation interventions was determined. Using the GRADE approach, the certainty was evaluated. I^2^ statistics and sensitivity analysis were utilized to measure heterogeneity. Egger’s test and funnel plot analysis were used to assess publication bias. **Results:** The pregnant women who received cognitive-behavioral counseling and financial incentives recognized a significant rise in their smoking abstinence rate (RR: 1.14, 95% CI: 1.02–1.28, *p* = 0.03 and RR: 2.37, 95% CI: 1.92–2.93, *p* < 0.001), but there was no significant difference observed among other behavioral approaches or pharmaceutical therapy. Fetuses born to women in the intervention group had significantly larger birth weights (MD = 94.73, 95% CI = (41.18–58.27), *p* < 0.001. **Conclusions:** Pregnant women respond well to cognitive-behavioral counseling and financial incentives for quitting smoking, which improve pregnancy outcomes like birth weight.

## 1. Introduction

Smoking is a global public health problem that has established adverse health effects on the whole population and pregnant individuals in particular [1,2]. Tobacco use during pregnancy is associated with serious maternal and fetal effects, such as ectopic pregnancy, placental abruption, miscarriage, stillbirth, preterm labor, low birth weight, and sudden infant death syndrome (SIDS) [3,4,5]. The consequences of maternal smoking are not limited only to short-term effects. It extends to negative long-term impacts on infants that may extend to adult life, such as obesity, dyslipidemia, insulin resistance, a higher chance of developing asthma, chronic obstructive lung diseases (COPD), hypertension, arrhythmia, and poorer academic performance [6,7,8,9].

Various preclinical studies show that nicotine exposure during pregnancy may change the infant’s underlying brain circuitry, response to neurotransmitters, and brain volume, as well as increase the risk for negative physical and mental health outcomes like attention-deficit hyperactivity disorder (ADHD), anxiety, and depression [10].

Despite the significant efforts that have been undertaken to raise awareness about the consequences of tobacco smoking on pregnancy outcomes among pregnant women, a lot of pregnant women continue to be smokers [11]. According to a 2018 meta-analysis, more than half of smoking women continue to smoke while they are pregnant; the prevalence of maternal smoking was 1.7% worldwide, 5.9% in the USA, and 8% in Europe [12].

There are several interventions to encourage quitting smoking during pregnancy. These include pharmacological interventions, including nicotine replacement therapy (NRT), and antidepressants like bupropion and varenicline (Chantix). Although NRT is thought to be the safest of them all, with a lower risk than cigarettes [13], there is not enough data to determine whether using it while pregnant can harm the fetus over the short and long term, as well as a limited number of trials on other pharmaceutical therapies in pregnancy [14]. According to a Cochrane review, further studies are required to determine the safety and effectiveness of pharmacotherapy for quitting smoking during pregnancy [15].

Due to the lack of clarity surrounding pharmacological therapies for expectant mothers, alternative behaviorally based interventions have emerged. They include several forms, such as cognitive counseling based on the “5 As” (Ask, Advise, Assess, Assist, Arrange). Because it is brief, simple to use, and successful, the American College of Obstetricians and Gynecologists recommended the “5 As” as the smoking cessation intervention of choice in 2010 and advised its systematic implementation for every pregnant smoker [16]. Counseling is commonly integrated as a part of a multicomponent cessation intervention for pregnant smokers, but the solitary impact of counseling is still unknown, and the results of individual randomized controlled trials (RCTs) are conflicting [17]. The emergence of digital platforms, especially after the year 2000, such as phone applications, software programs, and internet technology, facilitated the delivery of interventions by sending messages to pregnant individuals [18]. Among the benefits of digital interventions is that they can alter behavior and improve smoking cessation rates with respect to privacy and are accessible whenever needed [19]. Encouraging healthy lifestyle choices by applying a moderate level of physical activity program is advised during pregnancy and typically enhances the efficacy of behavioral support. However, there are not enough studies examining how physical activity affects quitting smoking [20]. Financial incentives are other forms of interventions directed at tobacco quitting during pregnancy. Incentives may be a promising technique to assist pregnant smokers in stopping smoking. Yet, this method has not been widely used in clinical practice, most likely due to the wide range of settings all over the world, intervention varieties, and the absence of definitive, conclusive research studies [21].

As an attempt to address the disparities in the evidence regarding the effectiveness of using various interventions to quit smoking during pregnancy, this study compared pharmacological and behavioral interventions with usual prenatal care, less intensive or different interventions, or a placebo for pregnant smokers in terms of smoking cessation and pregnancy outcomes.

## 2. Materials and Methods

To ensure integrity and adherence to best practices in methodology, this research was registered on the INPLASY database under registration number INPLASY202530054. Preferred Reporting Items for Systematic Review and Meta-Analysis (PRISMA) was adopted as the standard for reporting [22].

### 2.1. Searching Strategy

The researchers outline the eligibility requirements to select studies that address the following PICOs (patients, intervention, comparators, outcome, and study design) question. The authors searched for any randomized controlled trials (RCTs) in which pregnant women who were actively smoking cigarettes received any kind of smoking cessation intervention in comparison to usual prenatal care, less intensive or different interventions, or a placebo in pharmaceutical trials. The primary outcome was biochemically validated smoking abstinence at the end of pregnancy, and the outcomes of pregnancy were the secondary ones.

The authors searched the databases: PubMed, Cochrane Central Register of Controlled Trials (CENTRAL), Google Scholar, Science Direct, and International Clinical Trials Registry Platform (World Health Organization) from January 2010 to the end of December 2024. The following search terms were applied: “(smoking OR cigarette OR tobacco OR nicotine) AND (cessation OR quitting OR stopping OR giving up) AND (interventions OR programs OR trials OR strategies) AND (pregnancy OR pregnant OR gestation OR antenatal OR prenatal OR maternal)”.

### 2.2. Selection Criteria

#### 2.2.1. Inclusion Criteria

The researchers only considered free-whole text RCTs in English that included pregnant women at least eighteen years old who were actively smoking cigarettes and who were randomized to receive a smoking cessation intervention in the experimental group as opposed to a less intensive intervention, usual care, or a placebo in the control group.

#### 2.2.2. Exclusion Criteria

Manuscripts that are not RCTs, studies that involve pregnant non-smokers or passive smokers, studies that are not original research, and research that is published in languages other than English.

### 2.3. Quality Assessment

Separately, O.S.E., F.E.Y., and E.M.M. reviewed the literature, collected data, and evaluated the included studies for potential bias. If there was a disagreement, it was discussed and settled. The three reviewers combed through the literature, examining the abstract and title of each paper to weed out any that were blatantly improper, and then scanned the entire document to find the study that was appropriate for it. The majority of the extracted data fell into one of the following categories: (a) the literature’s essential elements, including country, the kind of smoking cessation intervention, sample size, first author, publication year, etc.; (b) the biochemically validated smoking abstinence rate; and (c) secondary outcomes, including the rate of cesarean sections, birth weight, stillbirth or miscarriage, preterm birth, and Apgar score < 7 (5 min).

### 2.4. Risk of Bias

Implementing the Cochrane risk-of-bias instrument for randomized trials (RoB 2) version 2 [23], three reviewers (O.S.E., F.E.Y., and N.MS.A.) evaluated the risk of bias in the chosen publications.

### 2.5. Statistical Analysis

The continuous and categorical dichotomous data were analyzed utilizing RevMan (Version 5.4.1; Cochrane Collaboration, Oxford, UK) to determine the pooled relative risk (RR) of various smoking cessation approaches among pregnant smokers. A model with random effects was employed for the meta-analysis to assume study-specific real effects based on heterogeneity. Significant heterogeneity is thought to be indicated by I^2^ values of more than 50% [24]. To determine how leaving out any intervention might affect the pooled effect estimates, a sensitivity analysis was carried out. Also, funnel plots [25] were applied to assess bias in publications, and the findings were verified by means of Egger’s regression asymmetry test [26]. Following the Grading of Recommendations Assessment, Development, and Evaluation (GRADE) technique, the degree of certainty was assessed [27]. By considering five domains—inconsistency, imprecision, indirectness, publication bias, and risk of bias—GRADE ratings of confidence are established. The degree of certainty was categorized as either very low, low, moderate, or high. When *p* values were less than 0.05, they were deemed significant.

## 3. Results

### 3.1. Features of the Research Involved

**Figure 1:**
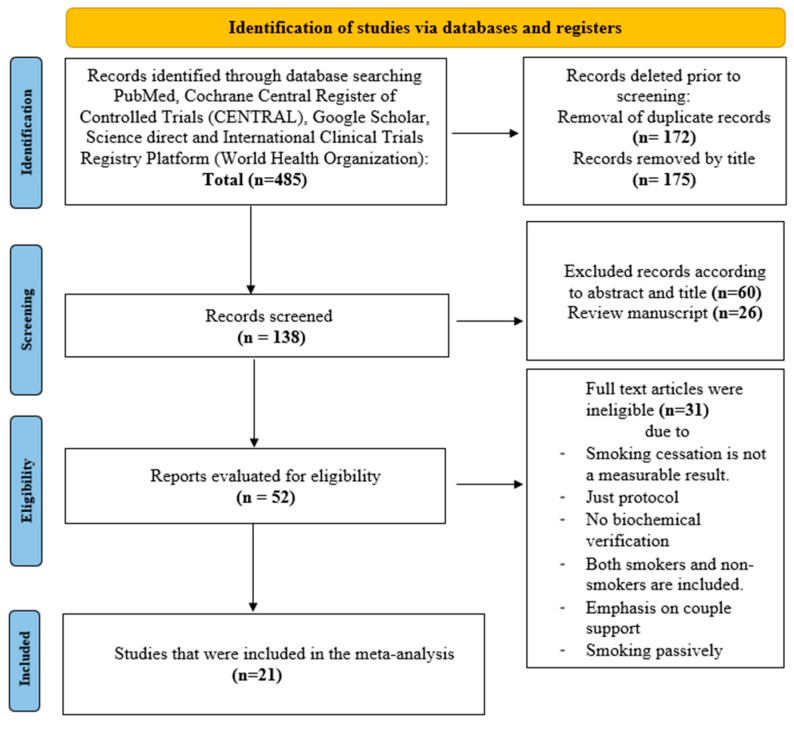
PRISMA search flow diagram.

This meta-analysis included 21 RCTs that met the inclusion and exclusion criteria, as presented in Figure 1. Their features, comprising the number of participants, the nation, and the intervention technique, are listed in Table 1. The pharmacological approach comprised eight randomized controlled trials with pregnant smokers (*n* = 2730), split into five for NRT [28,29,30,31,32] and three for bupropion [33,34,35]. The behavioral strategy encompassed thirteen RCTs with pregnant smokers (*n* = 5419), which were separated into three text message interventions [36,37,38], four financial incentive interventions [39,40,41,42], five cognitive-behavioral procedures [43,44,45,46,47], and one physical activity RCT [20].

### 3.2. Pharmacologic Interventions

**Figure 2:**
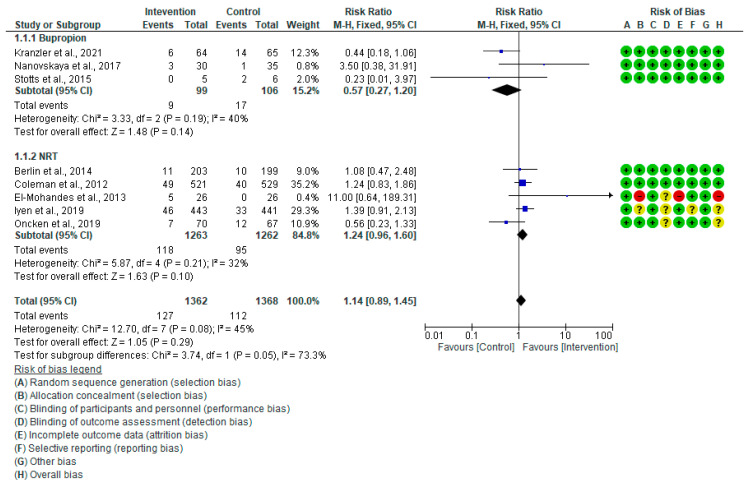
Forest plot of pharmacological smoking abstinence rate Kranzler et al., 2021 [35], Nanovskaya et al., 2017 [34], Stotts et al., 2015 [33], Berlin et al., 2014 [30], Coleman et al., 2012 [28], El-Mohandes et al., 2013 [29], Iyen et al., 2019 [31], Oncken et al., 2019 [32].

**Figure 3:**
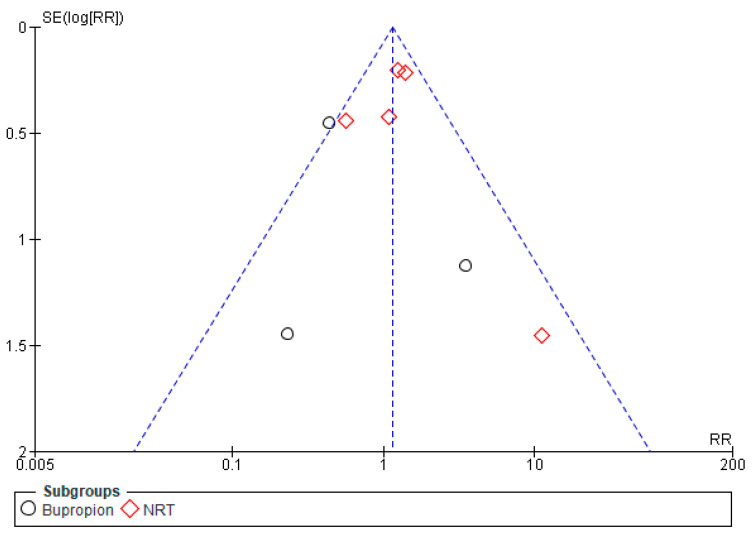
Funnel plot for pharmacological interventions.

Eight studies’ pooled results [28,29,30,31,32,33,34,35] showed inconclusive differences in smoking abstinence among the pregnant women in both pharmacological and placebo groups (Risk Ratio (RR): 1.14, 95% CI: 0.89–1.45, *p* = 0.29), as shown in Figure 2. According to subgroup analysis, the results from five studies [28,29,30,31,32] demonstrate that the NRT group’s smoking cessation was marginally higher than that of the placebo group; however, this difference was not statistically significant (RR: 1.24, 95% CI: 0.96–1.60, *p* = 0.10). The data from three studies [33,34,35] showed that bupropion had no significant effect on the intervention group’s smoking abstinence when compared to the control group (RR: 0.57, 95% CI: 0.27–1.20, *p* = 0.14) (Figure 2). The overall heterogeneity (I^2^) between the studies included in the analysis was 45%. Eliminating Nanovskaya et al. (2017) [34] reduced I^2^ in the sensitivity analysis for bupropion from 40% to 0%, while excluding Oncken et al. (2019) [32] lowered I^2^ in the sensitivity analysis for NRT from 32% to 0%. However, overall, I^2^ for pharmaceutical intervention remained unaltered. Egger’s test for bupropion and NRT (*p* = 0.47 and 0.80) and the funnel plot for pharmaceutical therapies, as shown in Figure 3, also demonstrated that publication bias was absent. NRT and bupropion have a moderate GRADE level of evidence (Supplementary File S1).

### 3.3. Behavioral Interventions

**Figure 4:**
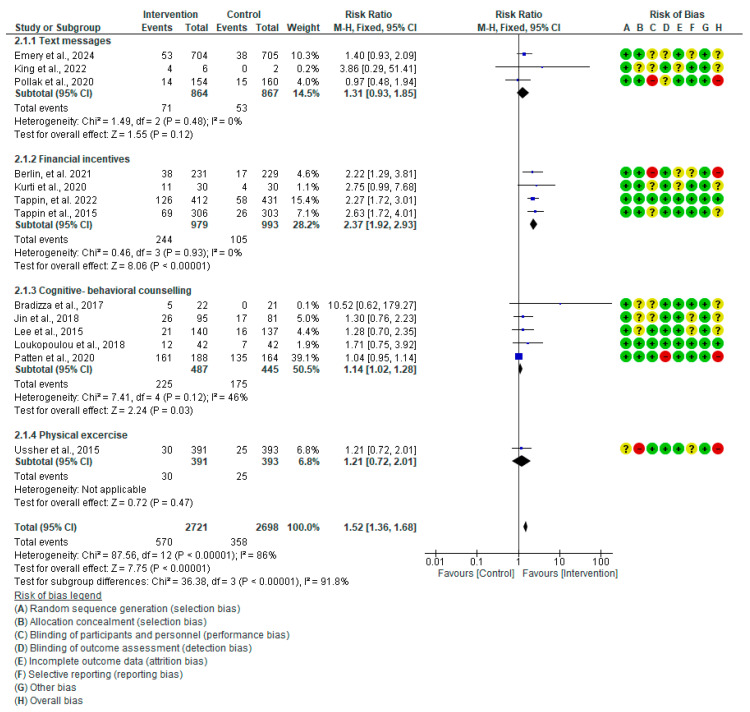
Forest plot of behavioral smoking abstinence rate Emery et al., 2024 [36], King et al., 2022 [37], Pollak et al., 2020 [38], Berlin et al., 2021 [39], Kurti 2020 [40], Tappin et al., 2022 [42], Tappin et al., 2015 [41], Bradizza et al., 2017 [43], Jin et al., 2018 [44], Lee et al., 2015 [45], Loukopoulou et al., 2018 [46], Patten et al., 2020 [47], Ussher et al., 2015 [20].

**Figure 5:**
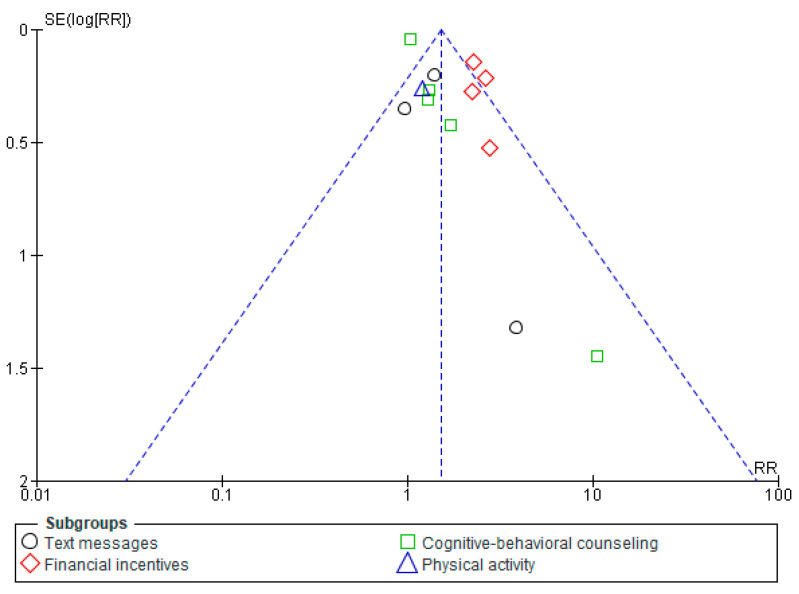
Funnel plot for behavioral interventions.

Thirteen RCTs [20,36,37,38,39,40,41,42,43,44,45,46,47] with a significant difference in smoking abstinence among pregnant women were included in a meta-analysis of all behavioral methods (RR: 1.52, 95% CI: 1.36–1.68, *p* < 0.001) (Figure 4). Subgroup analysis for each category reported that text message interventions [36,37,38] among pregnant smokers (*n* = 864) had no significant difference from controls (*n* = 867) in smoking quitting (RR: 1.31, 95% CI: 0.93–1.85, *p* = 0.12); however, pregnant smokers (*n* = 979) who received financial incentives [43,44,45,46] had a significant difference from controls (*n* = 993) (RR: 2.37, 95% CI: 1.92–2.93, *p* < 0.001). Additionally, a meta-analysis of cognitive-behavioral counseling [39,40,41,42,43] among pregnant smokers (*n* = 487) revealed a noteworthy distinction from controls (*n* = 445) (RR: 1.14, 95% CI: 1.02–1.28, *p* = 0.03). Interventions including text messages and financial incentives showed no heterogeneity (I^2^ = 0%), but cognitive-behavioral counseling showed moderate heterogeneity (I^2^ = 46%). One RCT for physical activity [20] was documented (RR: 1.21, 95% CI: 0.72–2.01, *p* = 0.47). Whereas the funnel plot for text messages and financial incentives showed a lack of publication bias (Egger’s test *p*-values = 0.69 and 0.74), it showed publication bias in cognitive-behavioral counseling studies (Egger’s test *p*-value = 0.003) (Figure 5). I^2^ dropped from 46% to 0% for the cognitive-behavioral therapy subgroup and from 86% to 40% for all behavioral therapies in the sensitivity analysis after deleting Patten et al. (2020) [47]. According to the GRADE level of certainty, financial incentives had a high degree, while text messages and cognitive-behavioral counseling had a moderate level, and physical exercise had a low one (Supplementary Files S1).

### 3.4. Pregnancy Outcomes

#### 3.4.1. Cesarean Section (CS)

**Figure 6:**
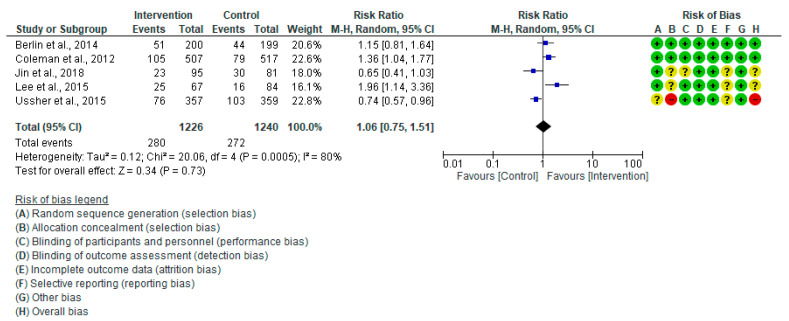
Forest plot of Caesarean section Berlin et al., 2014 [30], Coleman et al., 2012 [28], Jin et al., 2018 [44], Lee et al., 2015 [45], Ussher et al., 2015 [20].

When comparing the intervention group to the control group, the meta-analysis results of five studies [20,28,30,44,45] indicated no significant difference in the rate of CS (RR: 1.06, 95% CI = 0.75–1.51, *p* = 0.73) (Figure 6). A significant amount of variation existed among the studies included in this analysis (I^2^ = 80%, *p* = 0.0005). By eliminating Ussher et al. (2015) [20] in the sensitivity analysis, I^2^ dropped from 80% to 72%, but it did not drop to 0% by eliminating any studies.

#### 3.4.2. Birth Weight (gm)

**Figure 7:**
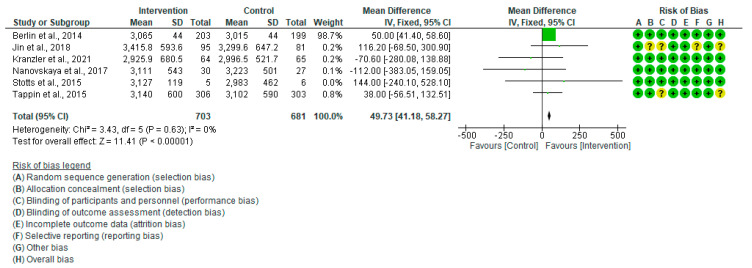
Forest plot of birth weight (gm) Berlin et al., 2014 [30], Jin et al., 2018 [44], Kranzler et al., 2021 [35], Nanovskaya et al., 2017 [34], Stotts et al., 2015 [33], Tappin et al., 2015 [41].

The pooled estimate of six trials [30,33,34,35,41,44] revealed that mothers in the intervention group gave birth to fetuses with significantly higher birth weight (MD = 94.73, 95% CI = (41.18–58.27), *p* = 0.000) (Figure 7). There was no significant variation among the trials included in this analysis (I^2^ = 0.0%, *p* = 0.63).

#### 3.4.3. Low Birth Weight (<2500 gm)

**Figure 8:**
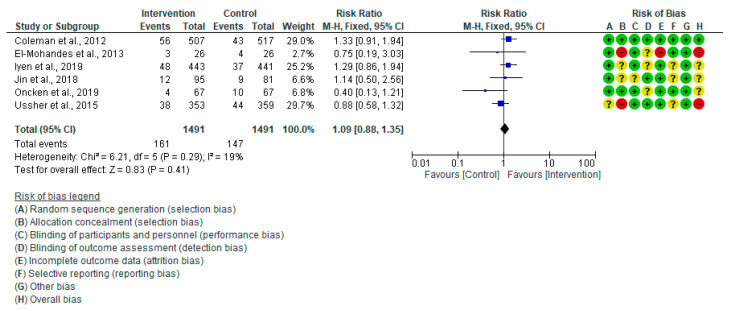
Forest plot of low birth weight: Coleman et al., 2012 [28], El-Mohandes et al., 2013 [29], Iyen et al., 2019 [31], Jin et al., 2018 [44], Oncken et al., 2019 [32], Ussher et al., 2015 [20].

The meta-analysis results of six studies [20,28,29,31,32,44] showed no evidence of a difference in the rate of low birth weight between the studied groups (RR: 1.09, 95% CI = 0.88–1.35, *p* = 0.41), and there was low, insignificant heterogeneity in the analysis (I^2^ = 19%, *p* = 0.29) (Figure 8). I^2^ declined from 19% to 0% in the sensitivity analysis when Oncken et al. (2019) [32] were eliminated.

#### 3.4.4. Apgar Score < 7 (5 Min)

**Figure 9:**
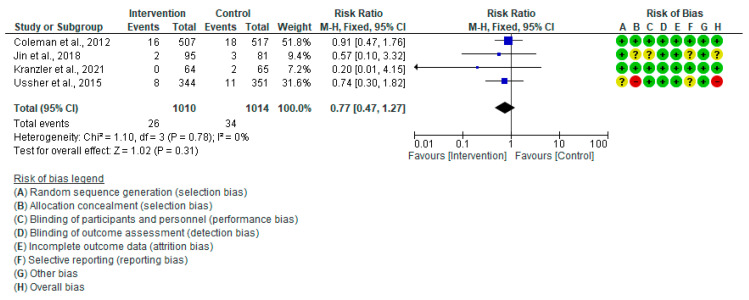
Forest plot of Apgar score < 7 (5 min): Coleman et al., 2012 [28], Jin et al., 2018 [44], Kranzler et al., 2021 [35], Ussher et al., 2015 [20].

The aggregated results of the four trial studies [20,28,35,44] demonstrated no significant difference in Apgar score < 7 (5 min) between the intervention and control groups (RR: 0.77, 95% CI = 0.47–1.27, *p* = 0.31), and there was no significant variation among the trials included in this analysis (I^2^ = 0.0%, *p* = 0.78). Therefore, fixed effect analysis was applied (Figure 9).

#### 3.4.5. Preterm Birth

**Figure 10:**
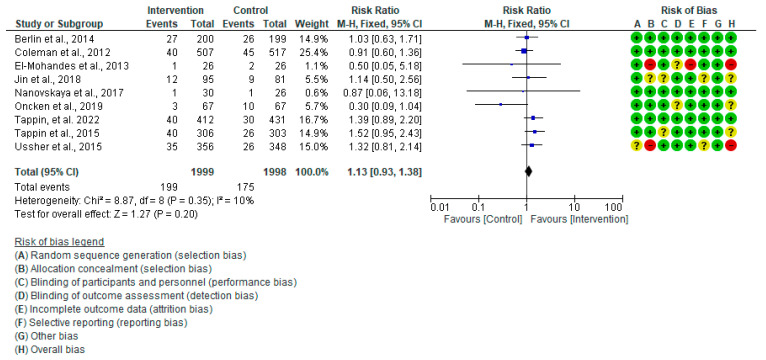
Forest plot of preterm birth: Berlin et al., 2014 [30], Coleman et al., 2012 [28], El-Mohandes et al., 2013 [29], Jin et al., 2018 [44], Nanovskaya et al., 2017 [34], Oncken et al., 2019 [32], Tappin et al., 2022 [42], Tappin et al., 2015 [41], Ussher et al., 2015 [20].

The meta-analysis of nine studies [20,28,29,30,32,34,41,42,44] found an inconclusive impact of the intervention or control measures on the incidence of preterm birth among the groups under study (RR: 1.13, 95% CI = 0.93–1.38, *p* = 0.20); there was low insignificant heterogeneity in the analysis (I^2^ = 10%, *p* = 0.35) (Figure 10). When Oncken et al. (2019) [32] were removed in the sensitivity analysis, I^2^ plummeted from 10% to 0%.

#### 3.4.6. Stillbirth

**Figure 11:**
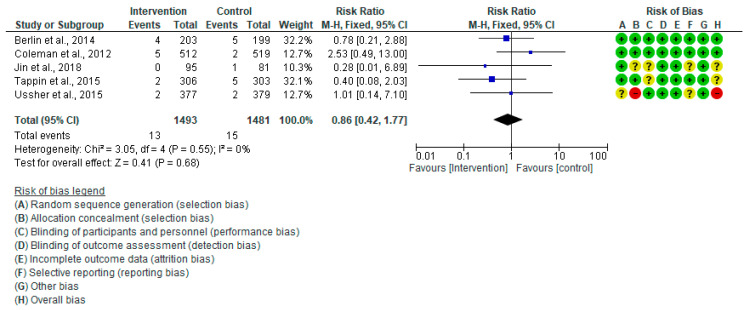
Forest plot of stillbirth: Berlin et al., 2014 [30], Coleman et al., 2012 [28], Jin et al., 2018 [44], Tappin et al., 2015 [41], Ussher et al., 2015 [20].

A meta-analysis of five RCTs [20,28,30,41,44] showed no significant consequence of the intervention on the study groups’ incidence of stillbirth (RR: 0.86, 95% CI = 0.42–1.77, *p* = 0.68). The analysis showed no heterogeneity (I^2^ = 0%) (Figure 11).

## 4. Discussion

Because of the well-established adverse health consequences of tobacco use during pregnancy, numerous therapies aimed at helping pregnant women quit smoking have been evaluated. The purpose of this study was to meta-analyze the results of RCTs from various databases to assess the impact of different smoking cessation strategies among pregnant smokers.

The smoking therapies included behavioral techniques like text messaging, financial incentives, cognitive-behavioral counseling, and physical activity, as well as pharmaceutical ones like bupropion or NRT. These clinical trials yielded the following extracted findings: the rate of cesarean sections, birth weight, stillbirth or miscarriage, premature delivery, Apgar score, and smoking cessation rate.

### 4.1. Pharmacological Interventions

Pregnant smokers who received pharmaceutical therapy, such as bupropion or NRT, were not significantly distinct from those who did not in terms of their smoking cessation rates or fetal outcomes, according to this meta-analysis of eight clinical trials. This agrees with the findings of Taylor et al. (2021) and Vila-Farinas et al. (2024), who reported that pregnant smokers do not respond well to pharmaceutical smoking cessation therapies [14,18]. However, a meta-analysis by Myung et al. (2012) found that medicine had a substantial impact on quitting smoking during pregnancy at the longest follow-up [48]. The use of pharmaceutical treatments to stop smoking during pregnancy is dubious due to the vulnerability of harmful consequences. Still, if the advantages-disadvantages balance of nicotine replacement during pregnancy is adequately assessed, it may be reasonable to use it [49].

### 4.2. Behavioral Interventions

There was a significant impact of behavioral therapies, according to the meta-analysis of the thirteen RCTs that were included in the behavioral approach. Subgroup meta-analysis of various behavioral interventions also revealed that the use of financial incentives and cognitive-behavioral counseling resulted in a considerable increase in smoking abstinence rates. This is consistent with research by Vila-Farinas et al. (2024), showing that financial incentives have the greatest percentage of quitting smoking, followed by electronic and counseling approaches among pregnant smokers [18]. Kock et al. (2023) and Notley et al. (2025) agreed that offering financial incentives to pregnant smokers increases their rates of quitting [50,51]. However, Griffiths et al. (2018) reported that behavior modification strategies, especially those based on computers and text-message initiatives, showed the biggest results [52]. According to a review by Lumley et al. (2009), promoting programs for quitting smoking during pregnancy lowered the percentage of smoking and had a significant pooled effect [53]. This significant role of behavioral intervention among pregnant smokers helps in quitting smoking in developing and developed countries.

### 4.3. Pregnancy Outcomes

Strategies for ceasing smoking increased the birth weight among pregnant women who received them, but they had no appreciable effect on the rate of cesarean sections, stillbirth, preterm delivery, or Apgar score, according to the pregnancy outcomes in this meta-analysis. In line with a review by Lumley et al. (2009), smoking cessation therapies during pregnancy improved the weight of the newborn and reduced preterm delivery [53]. Additionally, Taylor et al. (2021) evaluated NRT throughout pregnancy and found no proof of a change in the risk of miscarriage, stillbirths, or Apgar scores five minutes after delivery, according to RCT meta-analyses [14]. However, the birth weight pooling effect of the NRT group was much greater than that of the control group. There were no differences in birth weight or congenital malformations between the intervention and control groups, according to Turner et al.’s (2019) evaluation of bupropion [54].

### 4.4. Strengths and Limitations

The current meta-analysis has important strengths as it collects evidence from RCTS studies, which are initially classified at the top of the evidence hierarchy. To the best of our knowledge, this study is one of the few that investigates a variety of smoking cessation interventions and how they affect quitting smoking and pregnancy outcomes. Nevertheless, the limitations include a shortage of research on certain interventions, such as physical exercise, which makes it difficult to determine how this kind of intervention affects study results. Further research is necessary to explore the influence of a physical activity approach for smoking cessation. Additionally, there is not enough original research conducted in middle- and low-income countries to provide a complete picture of the various effects of programs in different populations with diverse healthcare settings, resources, and cultures.

### 4.5. Practical Implication

The findings of this study are important because they may be effectively applied in multiple clinical settings, where there are no formal programs for pregnant smokers to quit smoking. Cognitive-behavioral counseling, which is advised to be the first-line program for smoke cessation in pregnancy, may benefit pregnant smokers and their close family members, as well as the healthcare system, because treating the well-known negative health effects of maternal smoking during pregnancy would probably be more expensive than the cost of applying this smoking cessation intervention. Among the challenges to quitting smoking during pregnancy are social stigma, a lack of confidence and expertise, a sense of time constraints, and worries about destroying relationships. Nonetheless, it is recommended the presence of good communication at the institutional, social, and personal levels. Additionally, particular service structures and policies that encourage quitting smoking during pregnancy should be developed.

## 5. Conclusions

According to this meta-analysis’s findings, among different smoking cessation interventions, either pharmacological or behavioral approaches, financial incentives and cognitive-behavioral therapy were the most effective strategies that significantly promoted quitting smoking during pregnancy. The newborn’s mean weight was the most improved fetal outcome from smoking cessation interventions. However, further research on the efficacy, viability, and acceptability of various interventions in diverse healthcare service delivery settings across nations would be prudent. To ascertain the effectiveness of physical exercise regimens, the safety and efficacy of pharmaceutical therapies, and their long-term effects on fetal health, more research is also required.

## Figures and Tables

**Table 1:** Features of the research involved.

The Research Included	Region	Total Participants	Intervention Participants	Controls	Type of Intervention for Smoking Cessation	Extracted Outcomes	Limitations
Jin et al., 2018 [44]	China	176	95	81	Cognitive-behavioral counseling	Smoking abstinence, preterm birth, low birth weight, birth weight (gm), CS, stillbirth, Apgar score < 7 (5 min.)	- A short period of intervention. - No blinding of the participants.
Loukopoulou et al., 2018 [46]	Greece	84	42	42	Cognitive-behavioral counseling	Smoking abstinence	- Different week of gestation at enrolment between the groups.
Patten et al., 2020 [47]	USA	352	188	164	Behavioral counseling	Smoking abstinence	- Resources and logistic barriers for campaign delivery and follow-up of pregnant in rural areas.
Ussher et al., 2015 [20]	UK	784	391	393	Physical activity	Smoking abstinence, preterm birth, low birth weight, CS, stillbirth, Apgar score < 7 (5 min.)	- Difficult in groups’ allocation.
Kurti 2020 [40]	USA	60	30	30	Financial Incentives	Smoking abstinence	- No blinding of the participants.
Berlin et al., 2021 [39]	France	460	231	229	Financial Incentives	Smoking abstinence	- Lack of previous information on acceptability of financial incentives, and its cost-effectiveness.
Berlin et al., 2014 [30]	France	402	203	199	NRT	Smoking abstinence, preterm birth, birth weight (gm), CS, stillbirth	- Treatment started only after the end of the first trimester. - High nicotine dependant participants which may affect generalization for all pregnant smokers.
Coleman et al., 2012 [28]	UK	1050	521	529	NRT	Smoking abstinence, preterm birth, low birth weight, CS, stillbirth, Apgar score < 7 (5 min.)	Difficult in the generalization for low-income nations.
El-Mohandes et al., 2013 [29]	USA	52	26	26	NRT	Smoking abstinence, preterm birth, low birth weight	- Small sample size.
Oncken et al., 2019 [32]	USA	137	70	67	NRT	Smoking abstinence, preterm birth, low birth weight	- Low quitting rates in both groups leads to ending the trial prematurely.
Nanovskaya et al., 2017 [34]	USA	65	30	35	Bupropion	Smoking abstinence, preterm birth, birth weight (gm)	- The high early withdrawal rate.
Stotts et al., 2015 [33]	USA	11	5	6	Bupropion	Smoking abstinence, birth weight (gm),	- Small sample size - It was conducted in one urban clinic.
Kranzler et al., 2021 [35]	USA	129	64	65	Bupropion	Smoking abstinence, birth weight (gm), Apgar score < 7 (5 min.)	- Self-reporting was utilized for evaluating medication adherence, which could be overestimated.
Tappin et al., 2015 [41]	UK	609	306	303	Financial incentives	Smoking abstinence, preterm birth, birth weight (gm)	- It was conducted in one center.
Lee et al., 2015 [45]	USA	277	140	137	Cognitive-behavioral counseling	Smoking abstinence, CS	- Potential sample bias since low-income pregnant women did not receive prenatal care throughout the first trimester.
King et al., 2022 [37]	UK	28	15	13	Text-messaging	Smoking abstinence	Difficult in the recruitment to low socioeconomic pregnant women.
Emery et al., 2024 [36]	UK	1409	704	705	Text-messaging	Smoking abstinence	This intervention was effective only for short-term response.
Bradizza et al., 2017 [43]	USA	70	36	34	Emotion with cognitive-behavioral	Smoking abstinence	- Subject attrition during follow-ups. - Difficulties in conducting the extremely low-income participants.
Iyen et al., 2019 [31]	UK	884	443	441	NRT	Smoking abstinence, low birth weight,	Misattribution of smoking Status.
Pollak et al., 2020 [38]	USA	314	154	160	Text-messaging	Smoking abstinence	- Convenient sampling was collected which can affect the generalization of the results.
Tappin et al., 2022 [42]	UK	941	471	470	Financial incentives	Smoking abstinence, preterm birth	Difficult in the generalization for low-income nations.

NRT: Nicotine replacement therapy; CS: cesarean section.

## Supplementary Materials

The following are available online at https://www.mdpi.com/article/10.3390/healthcare13070732/s1, File S1. GRADE level of certainty; File S2. Risk of bias assessment; File S3. Pregnancy outcomes regarding types of interventions, File S4. PRISMA checklist.

## References

[1] Siddiqi K., Husain S., Vidyasagaran A., Readshaw A., Mishu M.P., Sheikh A. (2020). Global burden of disease due to smokeless tobacco consumption in adults: An updated analysis of data from 127 countries. BMC Med..

[2] Homa D.M., Neff L.J., King B.A., Caraballo R.S., Bunnell R.E., Babb S.D., Garrett B.E., Sosnoff C.S., Wang L. (2015). Centers for Disease Control and Prevention (CDC). Vital signs: Disparities in nonsmokers’ exposure to secondhand smoke—United States, 1999–2012. MMWR Morb Mortal Wkly Rep..

[3] Pineles B.L., Hsu S., Park E., Samet J.M. (2016). Systematic Review and Meta-Analyses of Perinatal Death and Maternal Exposure to Tobacco Smoke During Pregnancy. Am. J. Epidemiol..

[4] Fernandez-Rodriguez B., Gomez A.R., Jimenez Moreno B.S., de Alba C., Galindo A., Villalain C., Pallás C., Herraiz I. (2021). Smoking influence on early and late fetal growth. J. Perinat. Med..

[5] Shobeiri F., Jenabi E. (2017). Smoking and placenta previa: A meta-analysis. J. Matern. Fetal Neonatal Med..

[6] Míguez M.C., Pereira B. (2021). Effects of active and/or passive smoking during pregnancy and the postpartum period. Anales de Pediatría.

[7] Duan P., Wang Y., Lin R., Zeng Y., Chen C., Yang L., Yue M., Zhong S., Wang Y., Zhang Q. (2021). Impact of early life exposures on COPD in adulthood: A systematic review and meta-analysis. Respirology.

[8] Holbrook B.D. (2016). The effects of nicotine on human fetal development. Birth Defects Res. C Embryo Today.

[9] Clifford A., Lang L., Chen R. (2012). Effects of maternal cigarette smoking during pregnancy on cognitive parameters of children and young adults: A literature review. Neurotoxicol. Teratol..

[10] Wells A.C., Lotfipour S. (2023). Prenatal nicotine exposure during pregnancy results in adverse neurodevelopmental alterations and neurobehavioral deficits. Adv. Drug Alcohol. Res..

[11] Connolly N., Kelly D., O’Donnell P., Hyde S. (2024). Effectiveness of smoking cessation interventions in pregnant women attending primary care: A scoping review. BJGP Open.

[12] Lange S., Probst C., Rehm J., Popova S. (2018). National, regional, and global prevalence of smoking during pregnancy in the general population: A systematic review and meta-analysis. Lancet Glob. Health.

[13] Cressman A.M., Pupco A., Kim E., Koren G., Bozzo P. (2012). Smoking cessation therapy during pregnancy. Can. Fam. Physician.

[14] Taylor L., Claire R., Campbell K., Coleman-Haynes T., Leonardi-Bee J., Chamberlain C., Berlin I., Davey M.A., Cooper S., Coleman T. (2021). Fetal safety of nicotine replacement therapy in pregnancy: Systematic review and meta-analysis. Addiction.

[15] Claire R., Chamberlain C., Davey M.A., Cooper S.E., Berlin I., Leonardi-Bee J., Coleman T. (2020). Pharmacological interventions for promoting smoking cessation during pregnancy. Cochrane Database Syst. Rev..

[16] American College of Obstetricians and Gynecologists (2010). Committee Opinion No. 471. Smoking cessation during pregnancy. Obstet. Gynecol..

[17] Filion K.B., Abenhaim H.A., Mottillo S., Joseph L., Gervais A., O’Loughlin J., Paradis G., Pihl R., Pilote L., Rinfret S. (2011). The effect of smoking cessation counselling in pregnant women: A meta-analysis of randomised controlled trials. BJOG Int. J. Obstet. Gynaecol..

[18] Vila-Farinas A., Pérez-Rios M., Montes-Martinez A., Ruano-Ravina A., Forray A., Rey-Brandariz J., Candal-Pedreira C., Fernández E., Casal-Acción B., Varela-Lema L. (2024). Effectiveness of smoking cessation interventions among pregnant women: An updated systematic review and meta-analysis. Addict. Behav..

[19] Tombor I., Neale J., Shahab L., Ruiz M., West R. (2015). Healthcare providers’ views on digital smoking cessation interventions for pregnant women. J. Smok. Cessat..

[20] Ussher M., Lewis S., Aveyard P., Manyonda I., West R., Lewis B., Marcus B., Riaz M., Taylor A., Daley A. (2015). Physical activity for smoking cessation in pregnancy: Randomised controlled trial. BMJ.

[21] Higgins S.T., Washio Y., Lopez A.A., Heil S.H., Solomon L.J., Lynch M.E., Hanson J.D., Higgins T.M., Skelly J.M., Redner R. (2014). Examining two different schedules of financial incentives for smoking cessation among pregnant women. Prev. Med..

[22] Page M.J., McKenzie J.E., Bossuyt P.M., Boutron I., Hoffmann T.C., Mulrow C.D., Shamseer L., Tetzlaff J.M., Akl E.A., Brennan S.E. (2021). The PRISMA 2020 statement: An updated guideline for reporting systematic reviews. BMJ.

[23] Higgins J.P.T., Savović J., Page M.J., Elbers R.G., Sterne J.A.C., Higgins J.P.T., Thomas J., Chandler J., Cumpston M., Li T., Page M.J., Welch V.A. (2024). Chapter 8: Assessing risk of bias in a randomized trial [last updated October 2019]. Cochrane Handbook for Systematic Reviews of Interventions Version 6.5.

[24] Deeks J., Higgins J., Altman D., Higgins J., Green S. (2008). Analyzing data and undertaking meta-analyses. Cochrane Handbook for Systematic Reviews of Interventions Version 5.0.0.

[25] Duval S., Tweedie R. (2000). Trim and fill: A simple funnel-plot-based method of testing and adjusting for publication bias in meta-analysis. Biometrics.

[26] Egger M., Davey Smith G., Schneider M., Minder C. (1997). Bias in meta-analysis detected by a simple, graphical test. Br. Med. J. Clin. Res. Educ..

[27] Schünemann H.J., Higgins J.P.T., Vist G.E., Glasziou P., Akl E.A., Skoetz N., Guyatt G.H., Higgins J.P.T., Thomas J., Chandler J., Cumpston M., Li T., Page M.J., Welch V.A. (2024). Chapter 14: Completing ‘Summary of findings’ tables and grading the certainty of the evidence [last updated August 2023]. Cochrane Handbook for Systematic Reviews of Interventions Version 6.5.

[28] Coleman T., Cooper S., Thornton J.G., Grainge M.J., Watts K., Britton J., Lewis S. (2012). Smoking, Nicotine, and Pregnancy (SNAP) Trial Team. A randomized trial of nicotine-replacement therapy patches in pregnancy. N. Engl. J. Med..

[29] El-Mohandes A.A., Windsor R., Tan S., Perry D.C., Gantz M.G., Kiely M. (2013). A randomized clinical trial of trans-dermal nicotine replacement in pregnant African-American smokers. Matern. Child Health J..

[30] Berlin I., Grangé G., Jacob N., Tanguy M.L. (2014). Nicotine patches in pregnant smokers: Randomised, placebo controlled, multicentre trial of efficacy. BMJ.

[31] Iyen B., Vaz L.R., Taggar J., Cooper S., Lewis S., Coleman T. (2019). Is the Apparently Protective Effect of Maternal Nicotine Replacement Therapy (NRT) used in Pregnancy on Infant Development Explained by Smoking Cessation?: Secondary Analyses of a Randomised Controlled Trial. BMJ Open.

[32] Oncken C., Dornelas E.A., Kuo C.L., Sankey H.Z., Kranzler H.R., Mead E.L., Thurlow M.S.D. (2019). Randomized Trial of Nicotine Inhaler for Pregnant Smokers. Am. J. Obstet. Gynecol. MFM.

[33] Stotts A.L., Northrup T.F., Cinciripini P.M., Minnix J.A., Blalock J.A., Mullen P.D., Pedroza C., Blackwell S. (2015). Randomized, controlled pilot trial of bupropion for pregnant smokers: Challenges and future directions. Am. J. Perinatol..

[34] Nanovskaya T.N., Oncken C., Fokina V.M., Feinn R.S., Clark S.M., West H., Jain S.K., Ahmed M.S., Hankins G.D.V. (2017). Bupropion sustained release for pregnant smokers: A randomized, placebo-controlled trial. Am. J. Obstet. Gynecol..

[35] Kranzler H.R., Washio Y., Zindel L.R., Wileyto E.P., Srinivas S., Hand D.J., Hoffman M., Oncken C., Schnoll R.A. (2021). Placebo-controlled trial of bupropion for smoking cessation in pregnant women. Am. J. Obstet. Gynecol. MFM.

[36] Emery J., Leonardi-Bee J., Coleman T., McDaid L., Naughton F. (2024). The Effectiveness of Text Support for Stopping Smoking in Pregnancy (MiQuit): Multi-Trial Pooled Analysis Investigating Effect Moderators and Mechanisms of Action. Nicotine Tob. Res..

[37] King E., Cheyne H., Abhyankar P., Elders A., Grindle M., Hapca A., Jones C., O’Carroll R., Steele M., Williams B. (2022). Promoting smoking cessation during pregnancy: A feasibility and pilot trial of a digital storytelling intervention delivered via text-messaging. Patient Educ. Couns..

[38] Pollak K.I., Lyna P., Gao X., Noonan D., Bejarano Hernandez S., Subudhi S., Swamy G.K., Fish L.J. (2020). Efficacy of a Texting Program to Promote Cessation Among Pregnant Smokers: A Randomized Control Trial. Nicotine Tob. Res..

[39] Berlin I., Berlin N., Malecot M., Breton M., Jusot F., Goldzahl L. (2021). Financial incentives for smoking cessation in pregnancy: Multicentre randomised controlled trial. BMJ.

[40] Kurti A.N., Tang K., Bolivar H.A., Evemy C., Medina N., Skelly J., Nighbor T., Higgins S.T. (2020). Smartphone-based financial incentives to promote smoking cessation during pregnancy: A pilot study. Prev. Med..

[41] Tappin D., Bauld L., Purves D., Boyd K., Sinclair L., MacAskill S., McKell J., Friel B., McConnachie A., de Caestecker L. (2015). Cessation in Pregnancy Incentives Trial Team. Financial incentives for smoking cessation in pregnancy: Randomised controlled trial. BMJ.

[42] Tappin D., Sinclair L., Kee F., McFadden M., Robinson-Smith L., Mitchell A., Keding A., Watson J., Watson S., Dick A. (2022). Effect of financial voucher incentives provided with UK stop smoking services on the cessation of smoking in pregnant women (CPIT III): Pragmatic, multicentre, single blinded, phase 3, randomised controlled trial. BMJ.

[43] Bradizza C.M., Stasiewicz P.R., Zhuo Y., Ruszczyk M., Maisto S.A., Lucke J.F., Brandon T.H., Eiden R.D., Slosman K.S., Giarratano P. (2017). Smoking Cessation for Pregnant Smokers: Development and Pilot Test of an Emotion Regulation Treatment Supplement to Standard Smoking Cessation for Negative Affect Smokers. Nicotine Tob. Res..

[44] Jin G., Niu Y.Y., Yang X.W., Yang Y. (2018). Effect of smoking cessation intervention for pregnant smokers. Medicine.

[45] Lee M., Miller S.M., Wen K.Y., Hui S.K., Roussi P., Hernandez E. (2015). Cognitive-behavioral intervention to promote smoking cessation for pregnant and postpartum inner-city women. J. Behav. Med..

[46] Loukopoulou A.N., Vardavas C.I., Farmakides G., Rosolymos C., Chrelias C., Tzatzarakis M., Tsatsakis A., Myridakis A., Lyberi M., Behrakis P.K. (2018). Counselling for smoking cessation during pregnancy reduces tobacco-specific nitrosamine (NNAL) concentrations: A randomized controlled trial. Eur. J. Midwifery.

[47] Patten C.A., Lando H.A., Desnoyers C.A., Bock M.J., Alexie L., Decker P.A., Hughes C.A., Resnicow K., Burhansstipanov L., Boyer R. (2020). Healthy Pregnancies Project: Cluster Randomized Controlled Trial of a Community Intervention to Reduce Tobacco Use among Alaska Native Women. Int. J. Environ. Res. Public Health.

[48] Myung S., Ju W., Jung H., Park C., Oh S., Seo H., Kim H., Korean Meta-Analysis (KORMA) Study Group (2012). Efficacy and safety of pharmacotherapy for smoking cessation among pregnant smokers: A meta-analysis. BJOG Int. J. Obstet. Gynaecol..

[49] Morales-Suárez-Varela M., Puig B.M., Kaerlev L., Peraita-Costa I., Perales-Marín A. (2022). Safety of Nicotine Replacement Therapy during Pregnancy: A Narrative Review. Int. J. Environ. Res. Public Health.

[50] Notley C., Gentry S., Livingstone-Banks J., Bauld L., Perera R., Conde M., Hartmann-Boyce J. (2025). Incentives for smoking cessation. Cochrane Database Syst. Rev..

[51] Kock L.S., Erath T.G., Coleman S.R.M., Higgins S.T., Heil S.H. (2023). Contingency management interventions for abstinence from cigarette smoking in pregnancy and postpartum: A systematic review and meta-analysis. Prev. Med..

[52] Griffiths S.E., Parsons J., Naughton F., Fulton E.A., Tombor I., Brown K.E. (2018). Are digital interventions for smoking cessation in pregnancy effective? A systematic review and meta-analysis. Health Psychol. Rev..

[53] Lumley J., Chamberlain C., Dowswell T., Oliver S., Oakley L., Watson L. (2009). Interventions for promoting smoking cessation during pregnancy. Cochrane Database Syst. Rev..

[54] Turner E., Jones M., Vaz L.R., Coleman T. (2019). Systematic Review and Meta-Analysis to Assess the Safety of Bupropion and Varenicline in Pregnancy. Nicotine Tob. Res..

