# Supplementary material for: Breaking the Habit: A Systematic Review and Meta-Analysis of Pregnancy-Related Smoking Cessation Randomized Controlled Trials
Source: Healthcare (Basel). 2025 Mar 26;13(7):732. doi: 10.3390/healthcare13070732 (PMC11988373; doi:10.3390/healthcare13070732)
Supplement: Supplementary file 1 [file healthcare-13-00732-s001.zip › Supplementary File S1.pdf]

Outcome: smoking abstinence rate

Certainty assessment							№ of patients		Certainty
**Intervention**	**№ of studies**	**Risk of bias**	**Inconsistency**	**Indirectness**	**Imprecision**	**Others (Publication Bias)**	**intervention**	**control**	
**Bupropion**	3 RCTs	Not serious	Not serious	Not serious	Serious [a]	None	9/99 (9.1%)	17/106 (16.0%)	⊕⊕⊕◯ Moderate [a]
**NRT**	5 RCTs	Not serious	Not serious	Not serious	Serious [a]	None	118/1263 (9.3%)	95/1262 (7.5%)	⊕⊕⊕◯ Moderate [a]
**Text messages**	3 RCTs	Not serious	Not serious	Not serious	Serious [a]	None	71/864 (8.2%)	53/867 (6.1%)	⊕⊕⊕◯ Moderate [a]
**Financial incentives**	4 RCTs	Not serious	Not serious	Not serious	Not serious	None	244/979 (24.9%)	105/993 (10.6%)	⊕⊕⊕⊕ High
**Cognitive-behavioral counselling**	5 RCTs	Not serious	Not serious	Not serious	Not serious	Publication bias strongly suspected [b]	225/487 (46.2%)	175/445 (39.3%)	⊕⊕⊕◯ Moderate [b]
**Physical exercise**	1 RCTs	Serious	Not serious	Not serious	Serious [c]	None	30/391 (7.7%)	25/393 (6.4%)	⊕⊕◯◯ Low [c]

CI: confidence interval; **RR:** risk ratio

GRADE Working Group grades of evidence:
High certainty: having a high degree of confidence that the actual effect is close to the effect estimate.
Moderate certainty: having a moderate level of confidence in the effect estimate. The true effect may differ considerably from the estimate, but it is likely to be close to it.
Low certainty: having little confidence in the effect estimate. The estimated effect may differ greatly from the actual effect
Very low certainty: having very little confidence in the effect estimate: The true effect is likely to be much different from the estimated effect

Explanations

a. Imprecision due to few events and wide confidence interval suggesting small sample size in some studies

b. Eagger's regression test show significance p value (<0.05)

c. Outcome is evaluated from only one study
