# Supplementary material for: Breaking the Habit: A Systematic Review and Meta-Analysis of Pregnancy-Related Smoking Cessation Randomized Controlled Trials
Source: Healthcare (Basel). 2025 Mar 26;13(7):732. doi: 10.3390/healthcare13070732 (PMC11988373; doi:10.3390/healthcare13070732)

Risk of bias summary

	Random sequence generation (selection bias)	Allocation concealment (selection bias)	Blinding of participants and personnel (performance bias)	Blinding of outcome assessment (detection bias)	Incomplete outcome data (attrition bias)	Selective reporting (reporting bias)	Other bias	Overall bias
Berlin, et al. 2021	+	+	−	+	?	?	+	−
Berlin et al., 2014	+	+	+	+	+	+	+	+
Bradizza et al., 2017	+	?	?	+	+	+	+	?
Coleman et al., 2012	+	+	+	+	+	+	+	+
El-Mohandes et al., 2013	+	−	+	?	−	+	+	−
Emery et al., 2024	+	+	?	?	+	?	+	?
Iyen et al., 2019	+	?	+	?	+	?	+	?
Jin et al., 2018	+	?	?	+	+	?	+	?
King et al., 2022	+	?	?	+	?	+	+	?
Kranzler et al., 2021	+	+	+	+	+	+	+	+
Kurti et al., 2020	+	+	?	+	?	+	+	?
Lee et al., 2015	+	?	+	+	+	?	+	?
Loukopoulou et al., 2018	+	+	+	+	+	+	+	+
Nanovskaya et al., 2017	+	+	+	+	+	+	+	+
Oncken et al., 2019	+	+	+	?	+	+	+	?
Patten et al., 2020	+	+	+	−	+	+	+	−
Pollak et al., 2020	+	+	−	?	+	+	+	−
Stotts et al., 2015	+	+	+	+	+	+	+	+
Tappin, et al. 2022	+	+	+	+	+	+	+	+
Tappin et al., 2015	+	+	?	+	+	+	+	?
Ussher et al., 2015	?	−	+	+	+	?	+	−

Risk of bias graph

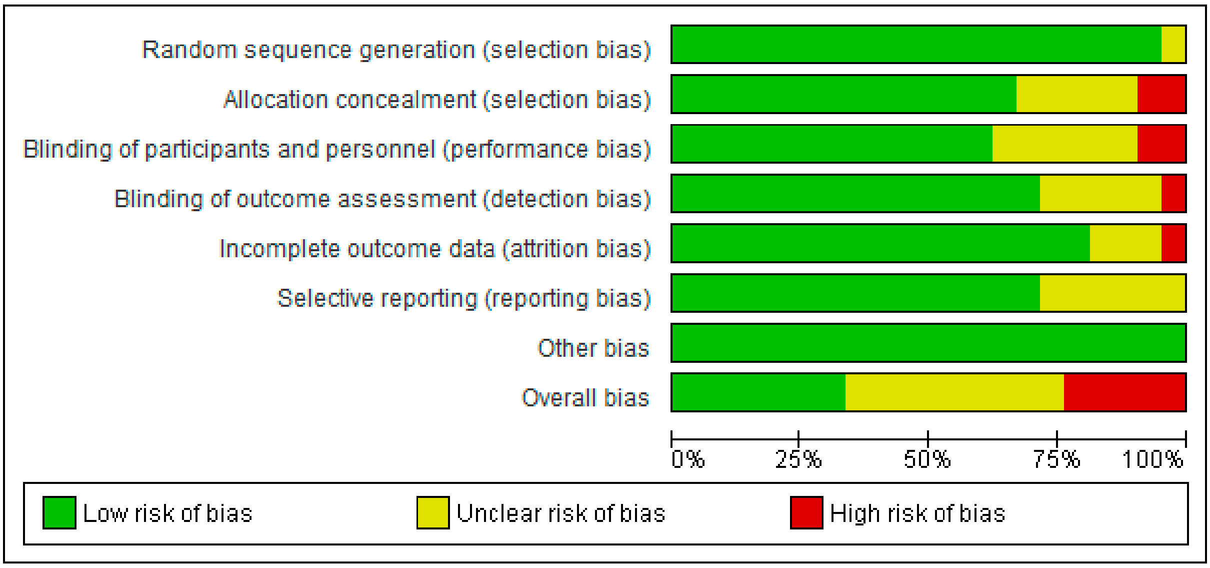

Supplement: Supplementary file 1 [file healthcare-13-00732-s001.zip › Supplementary File S2.pdf]
