# Supplementary material for: Breaking the Habit: A Systematic Review and Meta-Analysis of Pregnancy-Related Smoking Cessation Randomized Controlled Trials
Source: Healthcare (Basel). 2025 Mar 26;13(7):732. doi: 10.3390/healthcare13070732 (PMC11988373; doi:10.3390/healthcare13070732)
Supplement: Supplementary file 1 [file healthcare-13-00732-s001.zip › Supplementary File S3.pdf]

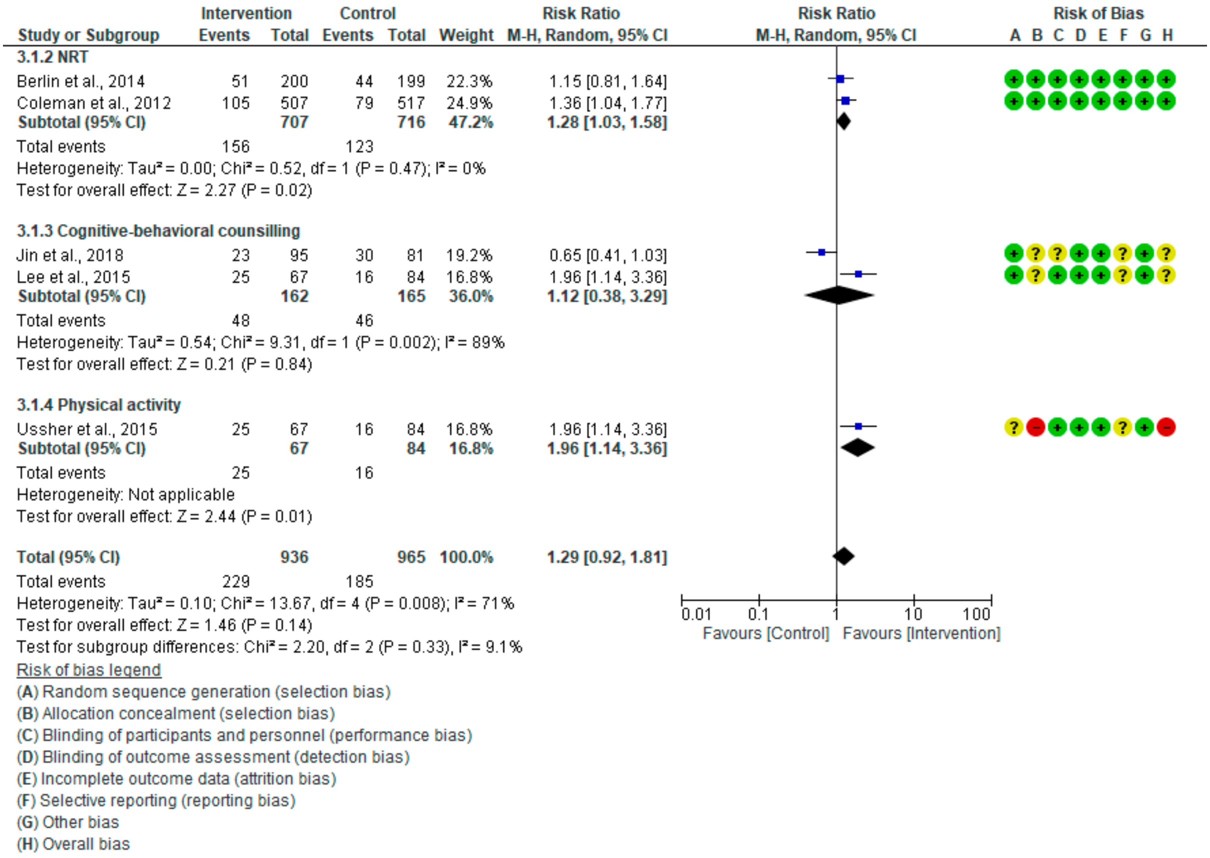

Cesarean section subgroup analysis regarding the smoking cessation interventions. Berlin et al., 2014 [30], Coleman et al., 2012 [28], Jin et al., 2018 [44], Lee et al., 2015 [45], Ussher et al., 2015 [20].

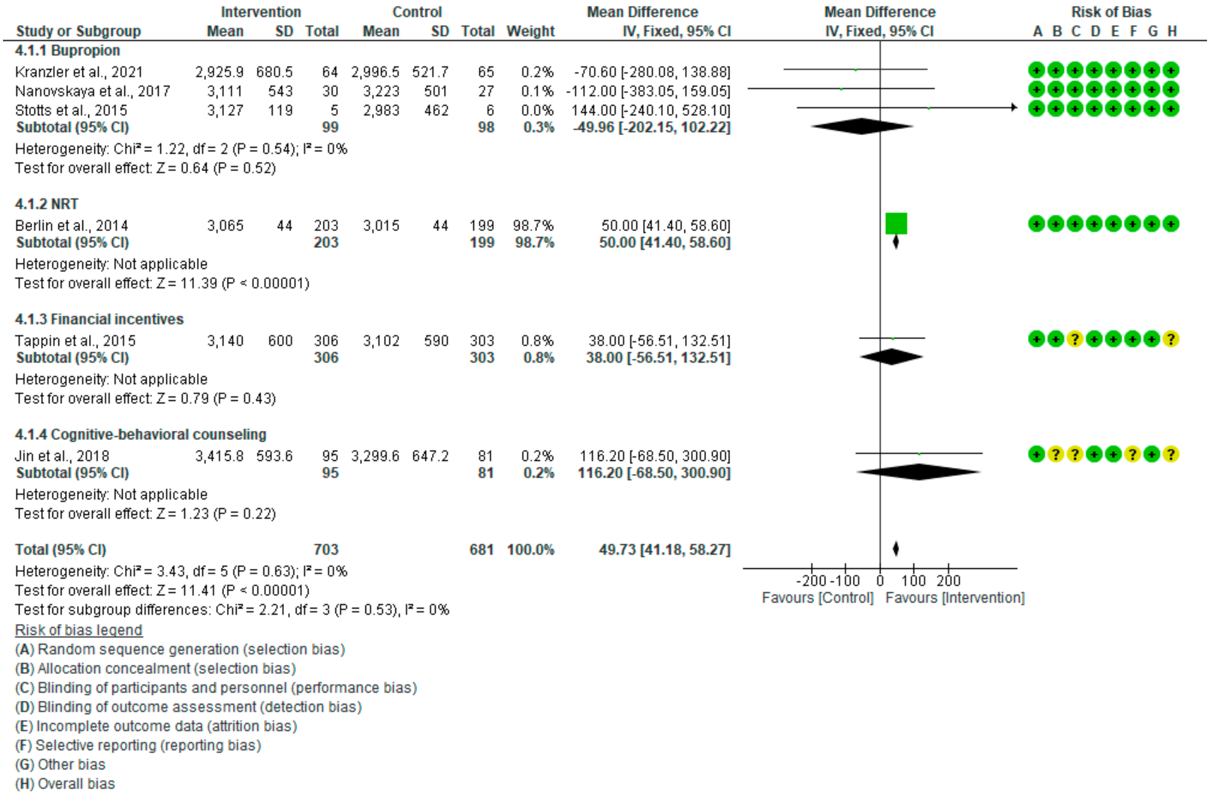

Birth weight (gm) subgroup analysis regarding the smoking cessation interventions. Kranzler et al., 2021 [35], Nanovskaya et al., 2017 [34], Stotts et al., 2015 [33], Berlin et al., 2014 [30], Tappin et al., 2015 [41], Jin et al., 2018 [44].

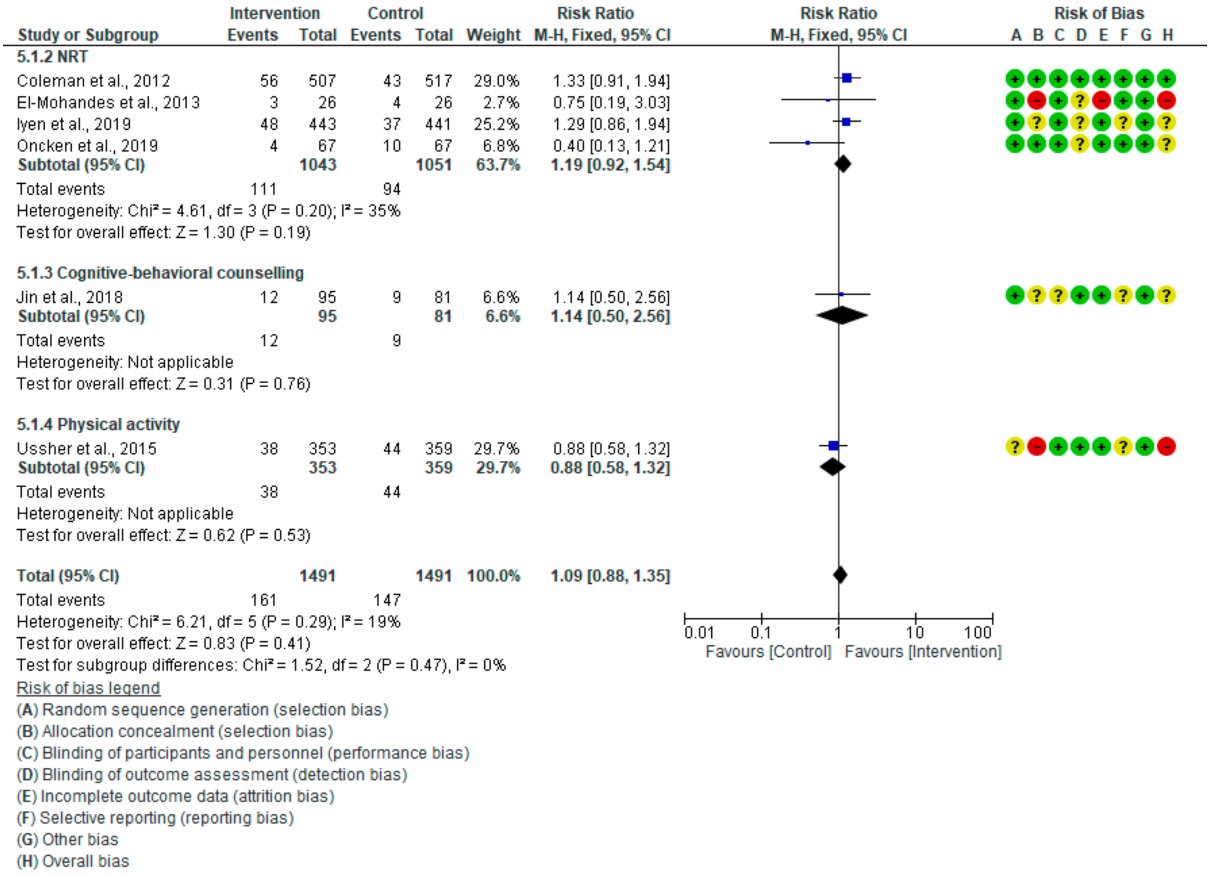

Low birth weight subgroup analysis regarding the smoking cessation interventions. Coleman et al., 2012 [28], El-Mohandes et al., 2013 [29], Iyen et al., 2019 [31], Oncken et al., 2019 [32], Jin et al., 2018 [44], Ussher et al., 2015 [20].

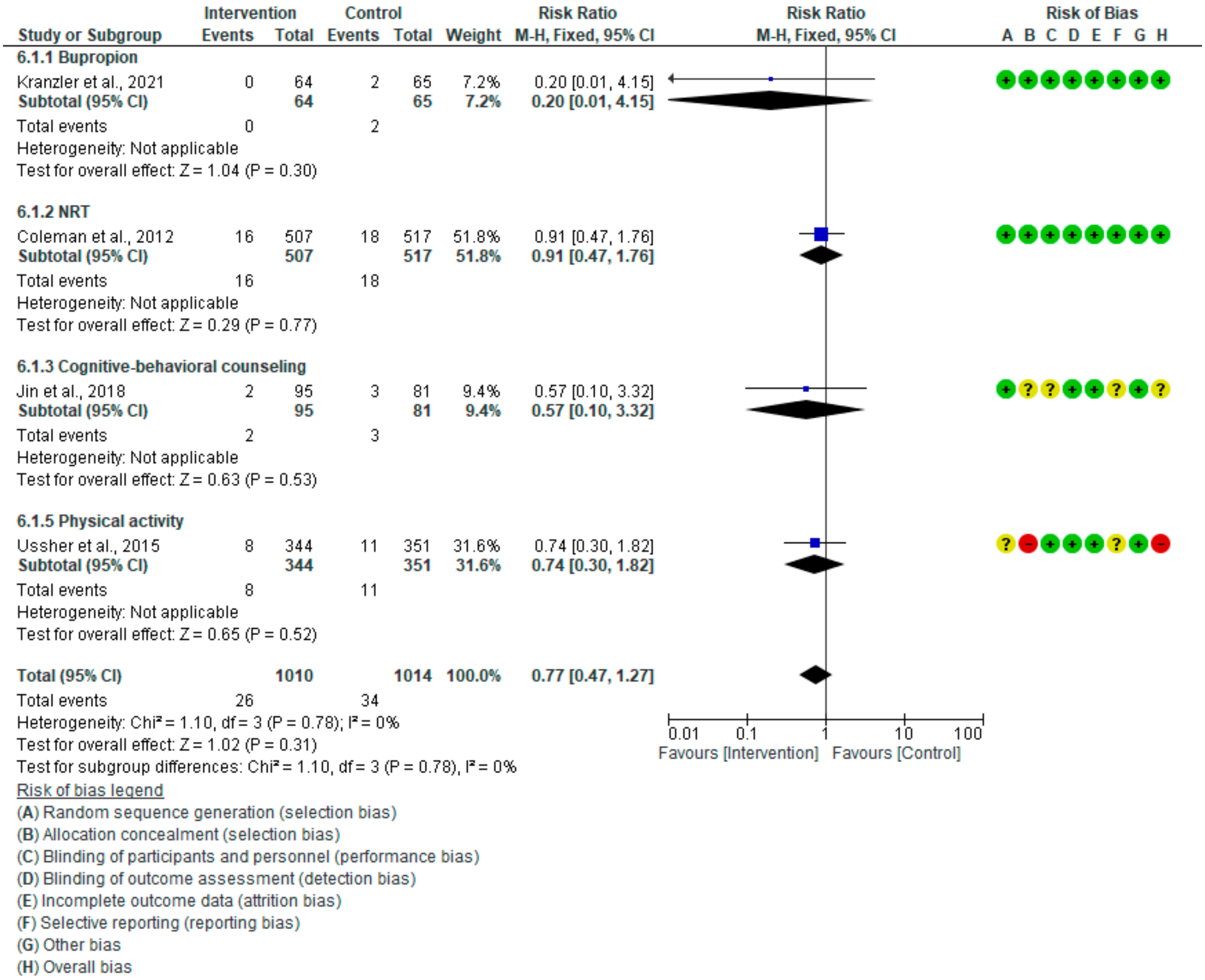

Apgar score<7(5 minutes) subgroup analysis regarding the smoking cessation interventions. Kranzler et al., 2021 [35], Coleman et al., 2012 [28], Jin et al., 2018 [44], Ussher et al., 2015 [20].

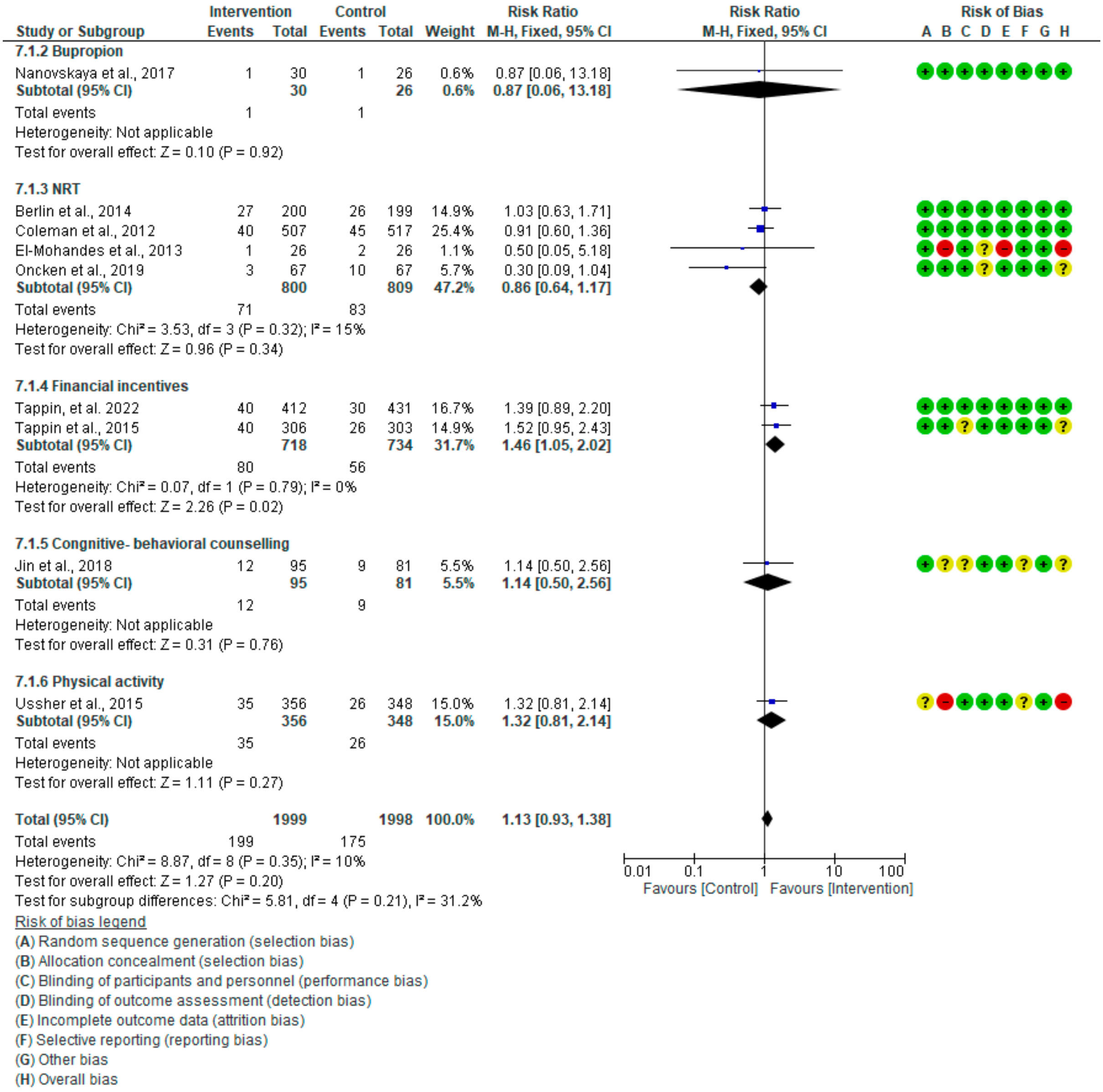

Preterm birth subgroup analysis regarding the smoking cessation interventions. Nanovskaya et al., 2017 [34], Berlin et al., 2014 [30], Coleman et al., 2012 [28], El-Mohandes et al., 2013 [29], Oncken et al., 2019 [32], Tappin et al., 2022 [42], Tappin et al., 2015 [41], Jin et al., 2018 [44], Ussher et al., 2015 [20].

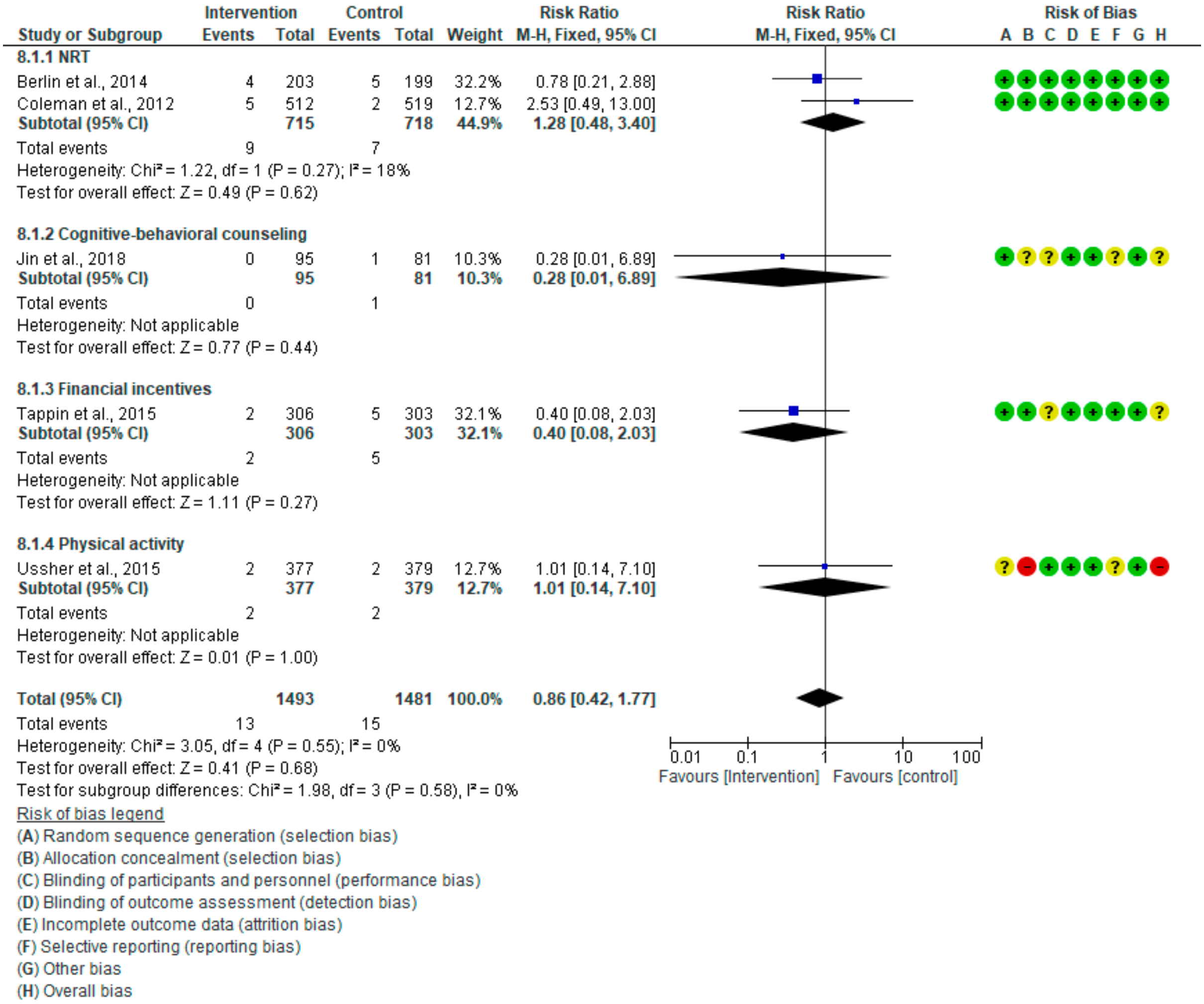

Stillbirth subgroup analysis regarding the smoking cessation interventions. Berlin et al., 2014 [30], Coleman et al., 2012 [28], Jin et al., 2018 [44], Tappin et al., 2015 [41], Ussher et al., 2015 [20].
